# Risk perception of the radiation health effects of decommissioning workers at Fukushima Daiichi Nuclear Power Plant

**DOI:** 10.1093/jrr/rraf064

**Published:** 2025-10-17

**Authors:** Hitomi Matsunaga, Aizhan Zabirova, Mengjie Liu, Yuya Kashiwazaki, Makiko Orita, Varsha Hande, Noboru Takamura

**Affiliations:** Department of Global Health, Medicine and Welfare, Atomic Bomb Disease Institute, Nagasaki University Graduate School of Biomedical Sciences, 1-12-4 Sakamoto, Nagasaki, 852-8523, Japan; Department of Global Health, Medicine and Welfare, Atomic Bomb Disease Institute, Nagasaki University Graduate School of Biomedical Sciences, 1-12-4 Sakamoto, Nagasaki, 852-8523, Japan; Department of Global Health, Medicine and Welfare, Atomic Bomb Disease Institute, Nagasaki University Graduate School of Biomedical Sciences, 1-12-4 Sakamoto, Nagasaki, 852-8523, Japan; Department of Global Health, Medicine and Welfare, Atomic Bomb Disease Institute, Nagasaki University Graduate School of Biomedical Sciences, 1-12-4 Sakamoto, Nagasaki, 852-8523, Japan; Department of Global Health, Medicine and Welfare, Atomic Bomb Disease Institute, Nagasaki University Graduate School of Biomedical Sciences, 1-12-4 Sakamoto, Nagasaki, 852-8523, Japan; City Cancer Challenge Foundation, 9 Rue du Commerce, 1204 Geneva, Switzerland; Department of Global Health, Medicine and Welfare, Atomic Bomb Disease Institute, Nagasaki University Graduate School of Biomedical Sciences, 1-12-4 Sakamoto, Nagasaki, 852-8523, Japan

To the Editors:

Fourteen years have passed since the Fukushima Daiichi Nuclear Power Plant (FDNPP) accident. During the FDNPP accident, a massive earthquake and tsunami caused the loss of the power supply needed for continuous cooling of the reactors. This led to a ‘meltdown’ in the three reactors that were in operation, producing fuel debris composed of melted nuclear fuel and surrounding structures. This highly radioactive fuel debris was estimated to weigh ~880 t. In November 2024, the Tokyo Electric Power Company (TEPCO) succeeded for the first time in experimentally collecting melted nuclear fuel debris from the accident after spending over 10 years on the effort [1]. The government of Japan and TEPCO are working towards completing decommissioning by 2051, 40 years or more after the accident. However, many of the processes have fallen behind the initial plans, making it unclear whether decommissioning can be completed as scheduled [2].

In connection with the treated water that began to be released into the ocean in 2023, the dismantling of several storage tanks, of more than 1000 originally built tanks, has begun, following the complete emptying of some units. At the site where the treated water tank was dismantled, facilities related to the full-scale removal of nuclear fuel debris are being constructed, and decommissioning work is still ongoing with extreme caution [3]. Decommissioning of the FDNPP is expected to take a long time. Hence, not only the training of engineers involved in the decommissioning process but also the management of their physical and mental health is one of the most important challenges.

This study investigated the risk perception of radiation health effects due to the decommissioning process of residents near the FDNPP. Residents in the area where the FDNPP is located are involved in its operations, and the progress of safe decommissioning is essential for the town’s reconstruction. The survey was conducted ~15 years after the FDNPP accident from November 2024 to January 2025. The questionnaire was developed based on the Fukushima Health Management Survey and previous cross-sectional studies conducted in Fukushima Prefecture [4, 5]. It included demographic information (e.g. age and sex, with age categorized into two groups: <65 and ≥65 years), whether they were interested in it, knowing how to obtain information about it and risk perception of radiation health effects due to the decommissioning process of the FDNPP. Two copies of the questionnaire were distributed to each household that held resident cards in Okuma Town (~5000) and Futaba Town (~2600), where the FDNPP is located, as well as in the neighboring town of Tomioka Town (~7200) in Fukushima Prefecture, at the time of the survey. Residents aged ≥18 years were asked to respond, and 1875 responses were collected: 704 from Tomioka, 702 from Okuma and 469 from Futaba. After excluding invalid responses, 1680 valid responses were included in the final analysis. Data was analyzed using SPSS software (Statistical Package for the Social Sciences) Version 29.0 (IBM, Armonk, NY, USA), with significance set at *P* < 0.05. This study was approved by the Ethics Committee of the Graduate School of Biomedical Sciences of Nagasaki University (approval no.: 24072602).

The survey showed that 67.1% (1128) of all respondents thought that workers involved in decommissioning the FDNPP would experience health effects due to radiation exposure. Interest in the decommissioning process of the FDNPP was extremely high, with 85.1% (1430) of the residents being interested in it. However, only 68.3% (1147) of respondents knew where to find information about decommissioning (Table 1).

**Table 1:** Comparisons of each factor of work experience related to the FDNPP

	Response	Overall % (*n*) (*n* = 1680)	Current employment % (*n*) 18.0 (303)	Past employment % (*n*) 34.5 (580)	No employment % (*n*) 47.5 (797)	*P*-value
Age (years)	<65	41.3 (694)	78.9 (239)	27.9 (162)	36.8 (293)	<0.01
	≥65	58.7 (986)	21.1 (64)	72.1 (418)	63.2 (504)	
Sex	Male	59.3 (996)	66.3 (201)	68.4 (397)	49.9 (398)	<0.01
	Female	40.7 (684)	33.7 (102)	31.6 (183)	50.1 (399)	
Offspring effects will occur because of the FDNPP accident	Yes	36.0 (604)	21.5 (65)	35.2 (204)	42.0 (335)	<0.01
	No	64.0 (1076)	78.5 (238)	64.8 (376)	58.0 (462)	
Health effects will occur because of working on FDNPP decommissioning	Yes	67.1 (1128)	45.2 (137)	64.0 (371)	77.8 (620)	<0.01
	No	32.9 (552)	54.8 (166)	36.0 (209)	22.2 (177)	
Interested in FDNPP decommissioning	Yes	85.1 (1430)	85.8 (260)	87.8 (509)	82.9 (661)	0.043
	No	14.9 (250)	14.2 (43)	12.2 (71)	17.1 (136)	
Knowing how to get information about FDNPP decommissioning	Yes	68.3 (1147)	85.1 (258)	71.9 (417)	59.2 (472)	<0.01
	No	31.7 (533)	14.9 (45)	28.1 (163)	40.8 (325)	

*Note.* Chi-squared test; FDNPP, Fukushima Daiichi Nuclear Power Plant.

Multinomial logistic regression analysis was performed with currently employed individuals as a reference, with adjustment for sex and age. Regarding the Model 1, the group that had never been involved in decommissioning work had an odds ratio (OR) of 1.84 (95% confidence interval (CI): 1.58–2.13, *P* < 0.01) of thinking that radiation health effects would occur because of the decommissioning process of the FDNPP compared with those currently employed. However, more of them did not know any sources of information about the decommissioning of the FDNPP compared with those currently employed (OR 2.95, 95% CI: 2.01–4.33, *P* < 0.01). Even in groups that had past employment in decommissioning, compared with those currently employed, the OR of thinking that radiation health effects would occur because of working on the decommissioning process of the FDNPP was 1.37 (95% Cl: 1.18–1.59, *P* < 0.01), whereas the OR of not knowing the information regarding the decommissioning of the FDNPP was 1.81 (95% CI: 1.21–2.70, *P* < 0.01) (model 1). It was also shown that, in both groups, the OR for the perception that radiation health effects may occur due to decommissioning work was higher than the perception that such effects may occur in children or grandchildren (model 1 vs model 2) (Table 2).

**Table 2:** Multinomial logistic regression results focused on work experience related to the FDNPP

Variable	Unit	Model 1: health effects		Model 2: offspring effects	
		OR (95% CI)		OR (95% CI)	
		Past employment	No employment	Past employment	No employment
Age (years)	<65/ ≥65	1.06^**^ (1.05–1.07)	1.05^**^ (1.04–1.06)	1.06^**^ (1.05–1.07)	1.05^**^ (1.04–1.06)
Sex	Male/Female	1.27 (0.92–1.76)	0.65^*^ (0.48–0.88)	1.18 (0.86–1.63)	0.58^**^ (0.43–0.79)
Interested in FDNPP decommissioning	Yes/No	1.16 (0.95–1.40)	1.16 (0.96–1.40)	1.14 (0.94–1.38)	1.11 (0.92–1.33)
Knowing how to get information about FDNPP decommissioning	No/Yes	1.81^*^ (1.21–2.70)	2.95^**^ (2.01–4.33)	1.89^*^ (1.27–2.83)	3.11^**^ (2.13–4.55)
Health effects will occur because of working on FDNPP decommissioning	Yes/No	1.37^**^ (1.18–1.59)	1.84^**^ (1.58–2.13)		
Offspring effects will occur because of FDNPP decommissioning	Yes/No			1.21^*^ (1.03–1.42)	1.43^**^ (1.23–1.67)

*Note*. Multinomial logistic regression analysis, reference: current employment. . ^*^P < 0.01, ^**^P < 0.001. OR, odds ratio; CI, confidence interval; FDNPP, Fukushima Daiichi Nuclear Power Plant; NA, not applicable

In the 2011 accident and emergency recovery work that immediately followed, <1% of all workers were exposed to external radiation >100 mSv [6]. Furthermore, estimates of the biological dose showed that both the internal and external exposure doses for individuals were limited, and it has been confirmed that no worker developed acute radiation syndrome [7, 8]. It is well known that health management for workers at the FDNPP was quite comprehensive, and great strategic efforts have been made not only to address the health effects of radiation exposure but also to manage various occupational risks such as heatstroke, mental stress, infectious diseases and job suitability [9]. In radiation management, the exposure dose limit for decommissioning workers is strictly regulated (50 mSv/year and 100 mSv/5 years), and comprehensive health management is being implemented, including regular health checkups, such as cataract and cancer screenings [10]. On the psychological side, it has become clear that workers have experienced severe psychological distress that has persisted over the long term, both as victims of the disaster and due to unknown recovery decommissioning work on the frontlines at the FDNPP [11]. Furthermore, it was evident that this psychological distress was exacerbated as workers involved in the FDNPP were viewed by the general public as perpetrators, leading to discrimination and slander, which led to further deterioration in their mental health [12]. With this background, the need for comprehensive mental health support for these workers has been strongly emphasized.

The most important finding of the study was that some people who had been involved in decommissioning work in the past, or their families, believed that radiation health effects would affect them, their children or grandchildren because of the decommissioning process. Since the decommissioning process will take a long period, it will continue to involve many people, including overseas experts of the next generation in advanced AI and robotic technologies. This study proposes that it is necessary to make efforts to protect the dignity of people exposed to radiation health risks as they are involved in the decommissioning process of the FDNPP.
