# Correction to: Risk perception of the radiation health effects of decommissioning workers at Fukushima Daiichi Nuclear Power Plant

**DOI:** 10.1093/jrr/rrag072

**Published:** 2026-08-11

This is a correction to: Hitomi Matsunaga, Aizhan Zabirova, Mengjie Liu, Yuya Kashiwazaki, Makiko Orita, Varsha Hande, Noboru Takamura, Risk perception of the radiation health effects of decommissioning workers at Fukushima Daiichi Nuclear Power Plant, *Journal of Radiation Research*, Volume 66, Issue 6, November 2025, Pages 746–749, https://doi.org/10.1093/jrr/rraf064

In the originally published version of this manuscript, there was a typographical error in the second co-author’s name.

This has been corrected to read: “Aizhan Zabirova”.

